# Supplementary material for: A Humanized Yeast Phenomic Model of Deoxycytidine Kinase to Predict Genetic Buffering of Nucleoside Analog Cytotoxicity
Source: Genes (Basel). 2019 Sep 30;10(10):770. doi: 10.3390/genes10100770 (PMC6826991; doi:10.3390/genes10100770)
Supplement: Supplementary file 1 [file genes-10-00770-s001.zip › Additional_Files/Additional_File7_GOTermHeatmaps.zip!/Additional_File7_GOTermHeatmaps/DNA-dependent_ATPase_activity.pdf]

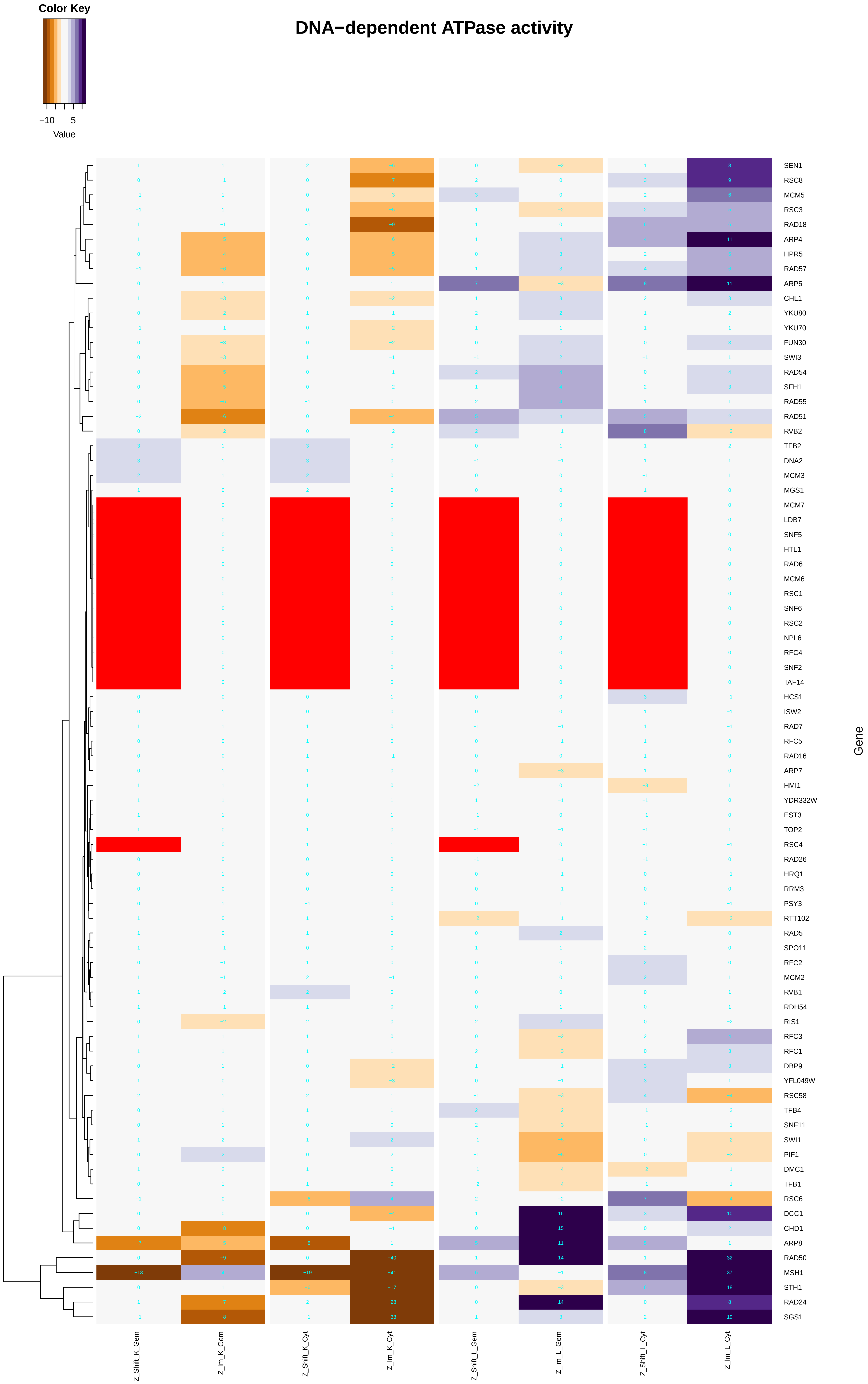
DNA−dependent ATPase activity
Color Key
−10
5
Value
Gene
SEN1
RSC8
MCM5
RSC3
RAD18
ARP4
HPR5
RAD57
ARP5
CHL1
YKU80
YKU70
FUN30
SWI3
RAD54
SFH1
RAD55
RAD51
RVB2
TFB2
DNA2
MCM3
MGS1
MCM7
LDB7
SNF5
HTL1
RAD6
MCM6
RSC1
SNF6
RSC2
NPL6
RFC4
SNF2
TAF14
HCS1
ISW2
RAD7
RFC5
RAD16
ARP7
HMI1
YDR332W
EST3
TOP2
RSC4
RAD26
HRQ1
RRM3
PSY3
RTT102
RAD5
SPO11
RFC2
MCM2
RVB1
RDH54
RIS1
RFC3
RFC1
DBP9
YFL049W
RSC58
TFB4
SNF11
SWI1
PIF1
DMC1
TFB1
RSC6
DCC1
CHD1
ARP8
RAD50
MSH1
STH1
RAD24
SGS1
Z_Shift_K_Gem
Z_Im_K_Gem
Z_Shift_K_Cyt
Z_Im_K_Cyt
Z_Shift_L_Gem
Z_Im_L_Gem
Z_Shift_L_Cyt
Z_Im_L_Cyt

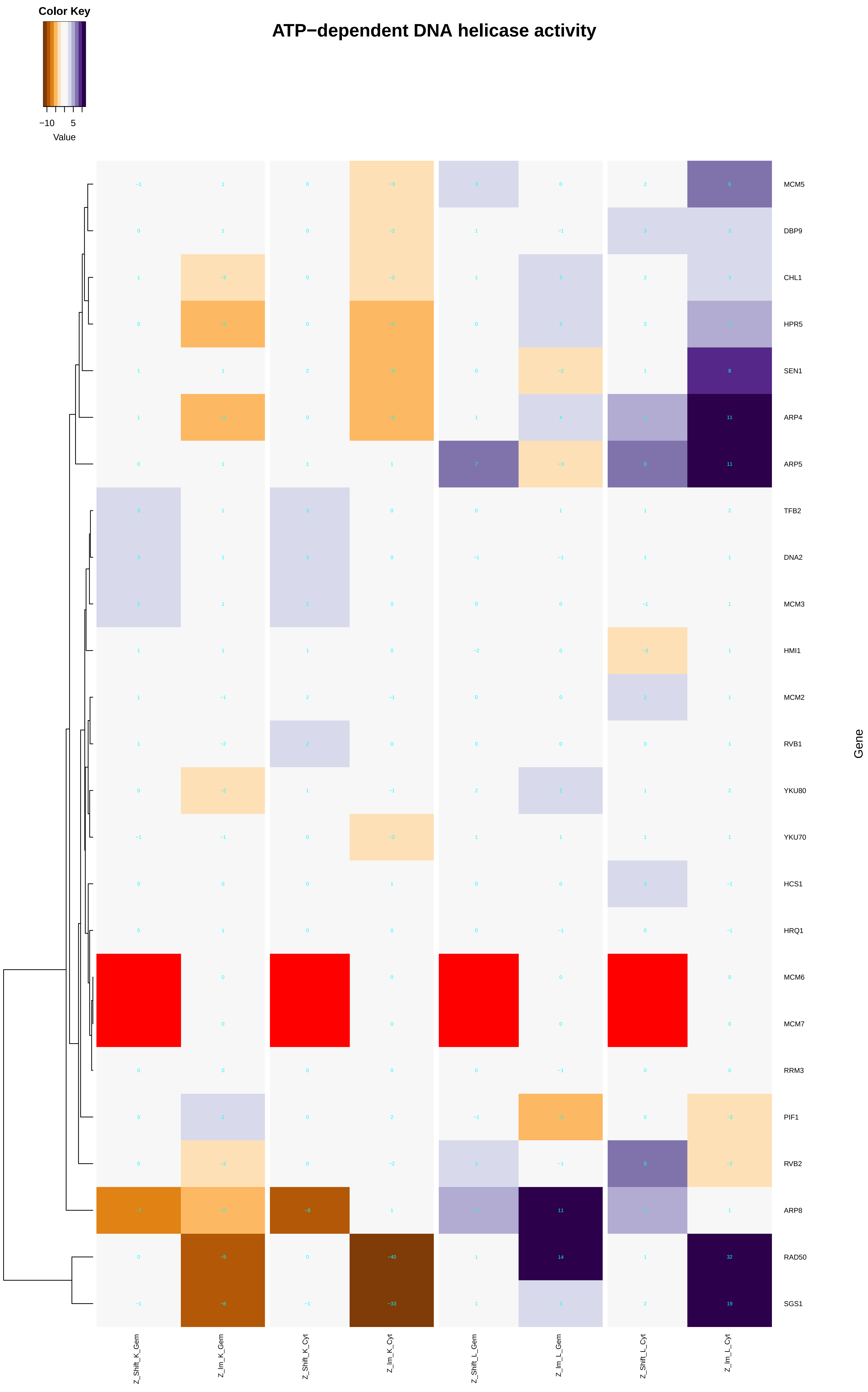
Color Key
−10 5
Value
ATP−dependent DNA helicase activity
MCM5
DBP9
CHL1
HPR5
SEN1
ARP4
ARP5
TFB2
DNA2
MCM3
HMI1
MCM2
RVB1
YKU80
YKU70
HCS1
HRQ1
MCM6
MCM7
RRM3
PIF1
RVB2
ARP8
RAD50
SGS1
Gene
Z_Shift_K_Gem
Z_Im_K_Gem
Z_Shift_K_Cyt
Z_Im_K_Cyt
Z_Shift_L_Gem
Z_Im_L_Gem
Z_Shift_L_Cyt
Z_Im_L_Cyt

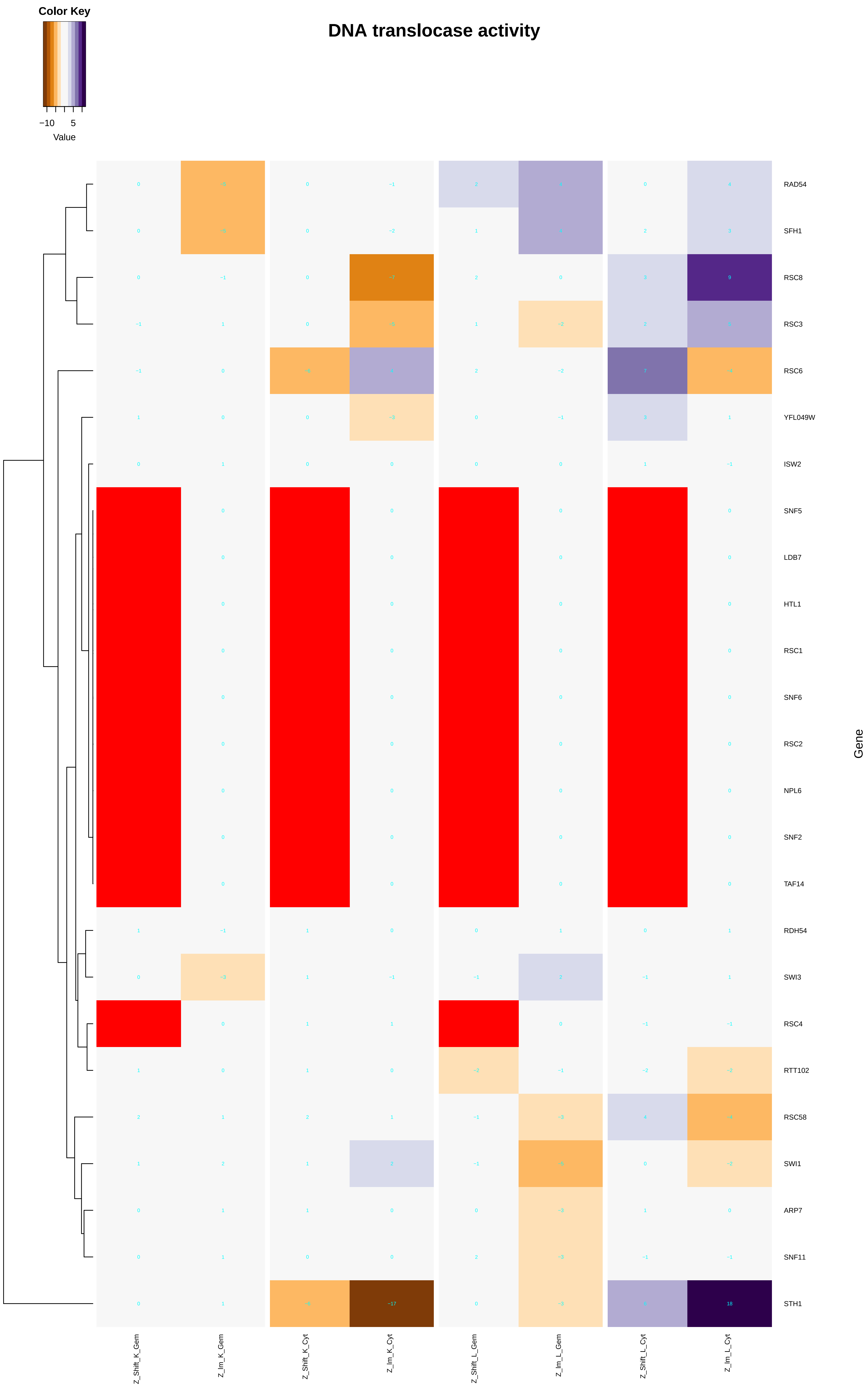
DNA translocase activity
Color Key
−10
5
Value
RAD54
SFH1
RSC8
RSC3
RSC6
YFL049W
ISW2
SNF5
LDB7
HTL1
RSC1
SNF6
RSC2
NPL6
SNF2
TAF14
RDH54
SWI3
RSC4
RTT102
RSC58
SWI1
ARP7
SNF11
STH1
Gene
Z_Shift_K_Gem
Z_Im_K_Gem
Z_Shift_K_Cyt
Z_Im_K_Cyt
Z_Shift_L_Gem
Z_Im_L_Gem
Z_Shift_L_Cyt
Z_Im_L_Cyt

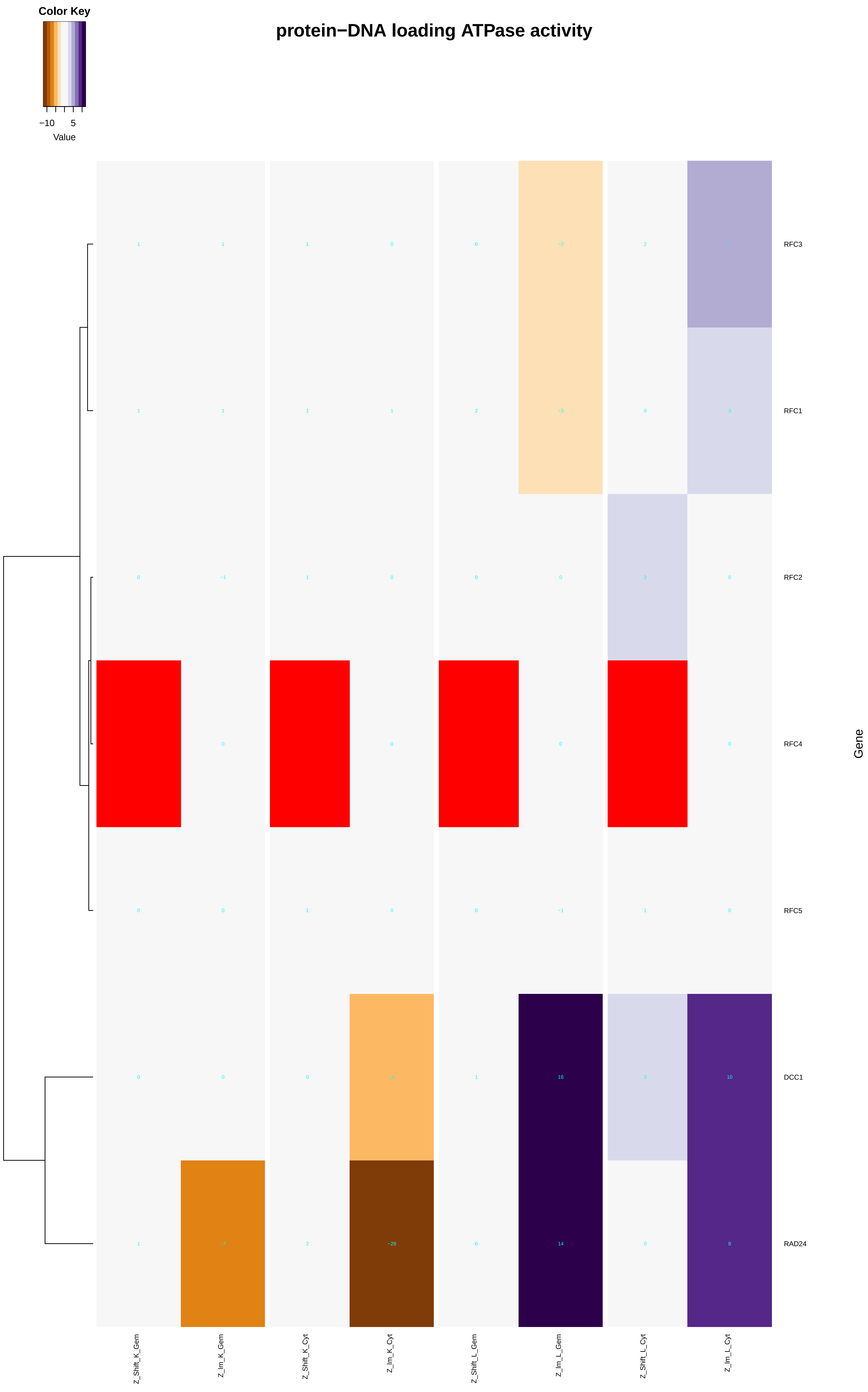
Color Key
−10
5
Value
protein−DNA loading ATPase activity
RFC3
RFC1
RFC2
RFC4
RFC5
DCC1
RAD24
Gene
Z_Shift_K_Gem
Z_Im_K_Gem
Z_Shift_K_Cyt
Z_Im_K_Cyt
Z_Shift_L_Gem
Z_Im_L_Gem
Z_Shift_L_Cyt
Z_Im_L_Cyt

ATP−dependent DNA/RNA helicase activity

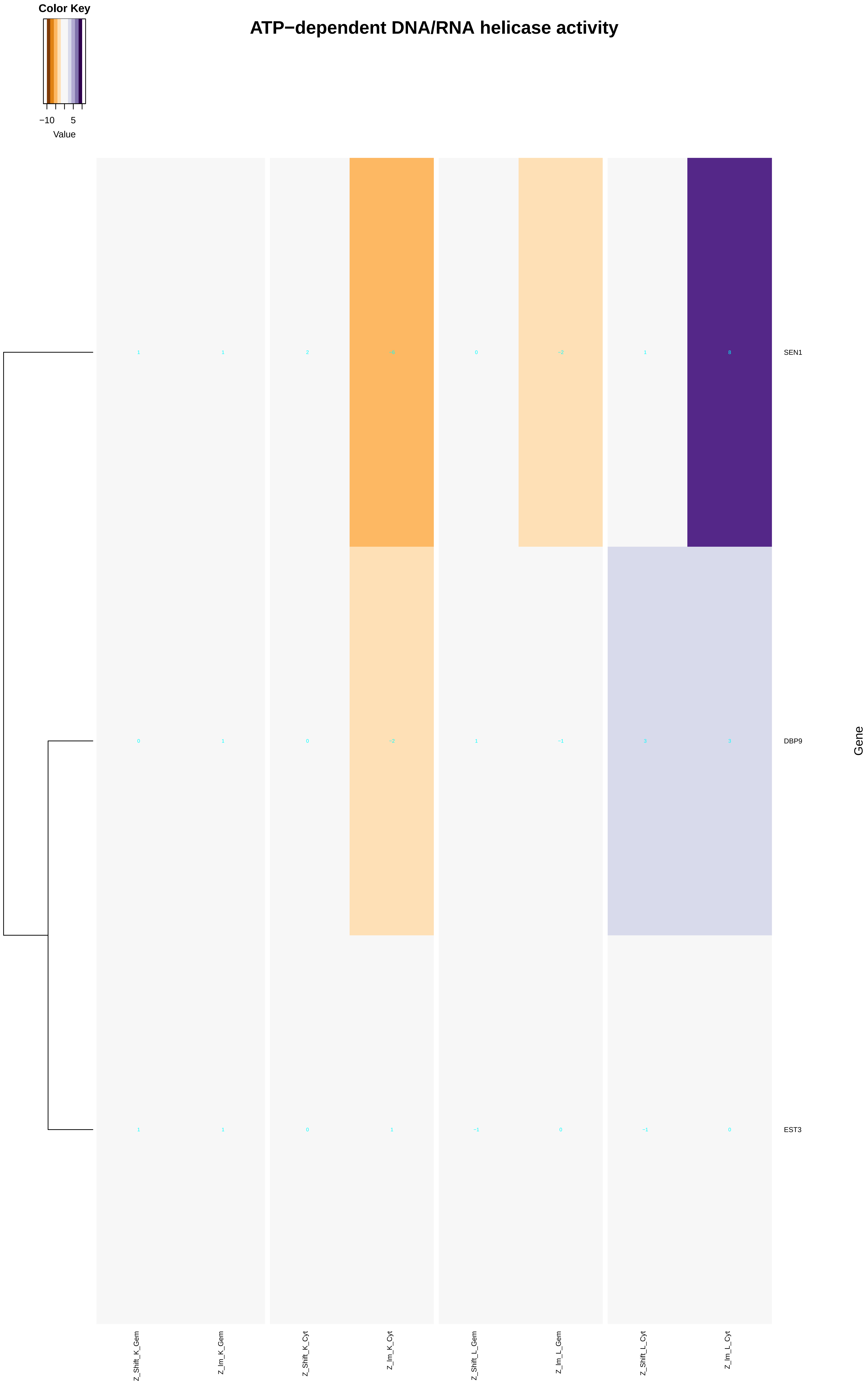

single−stranded DNA−dependent ATPase activity

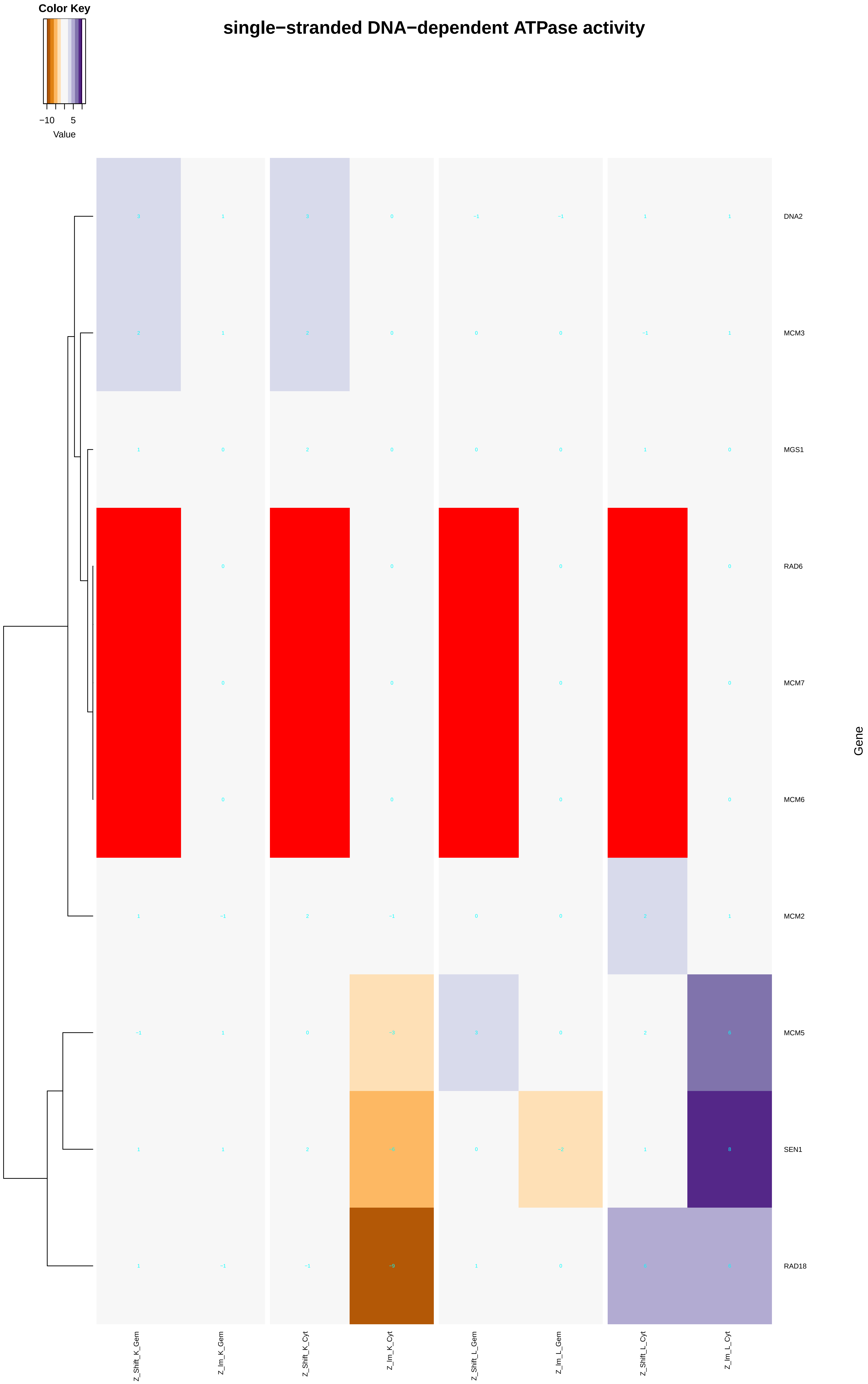

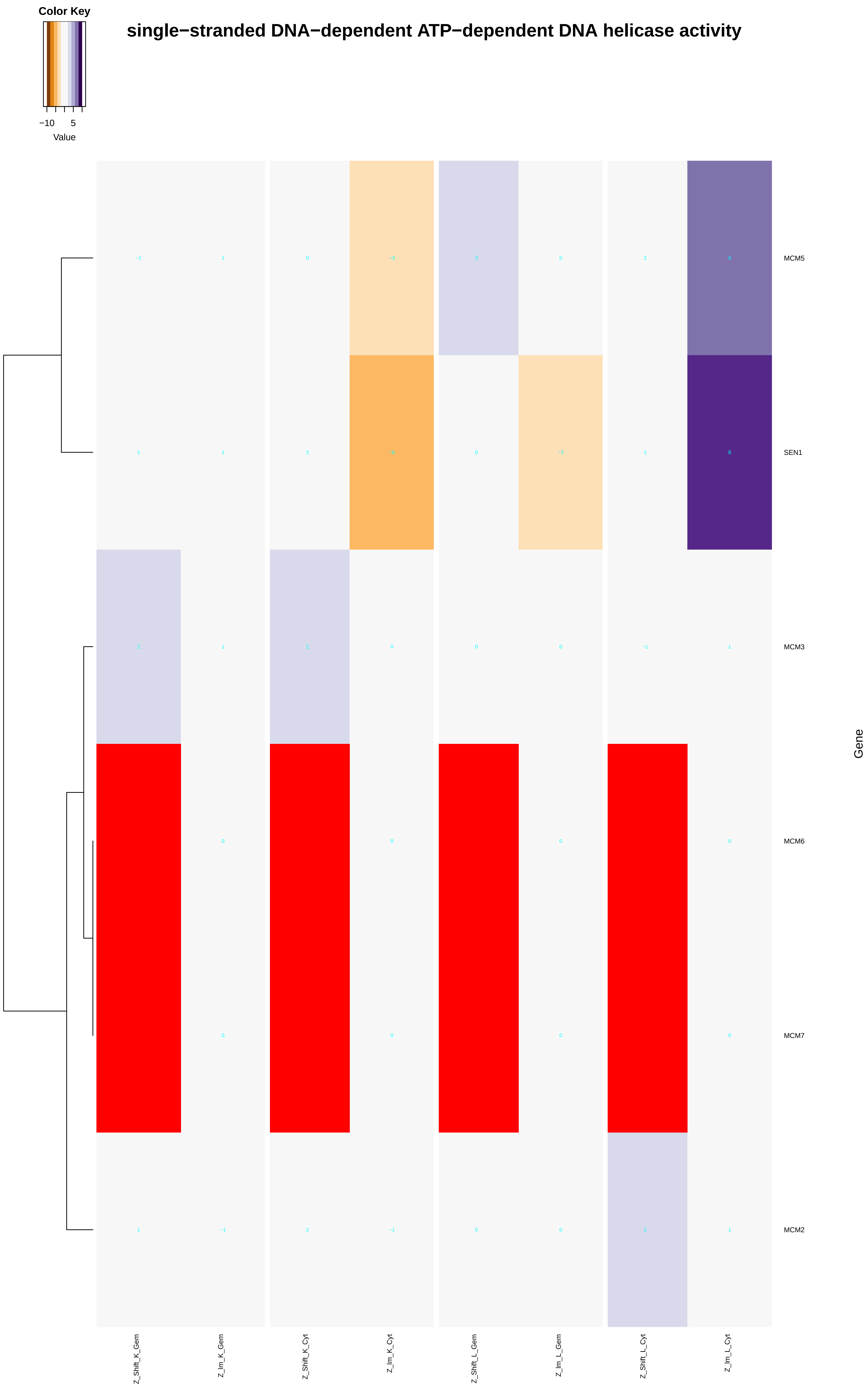
Color Key
−10
5
Value
single−stranded DNA−dependent ATP−dependent DNA helicase activity
MCM5
SEN1
MCM3
MCM6
MCM7
MCM2
Gene
Z_Shift_K_Gem
Z_Im_K_Gem
Z_Shift_K_Cyt
Z_Im_K_Cyt
Z_Shift_L_Gem
Z_Im_L_Gem
Z_Shift_L_Cyt
Z_Im_L_Cyt

ATP−dependent 3'−5' DNA helicase activity

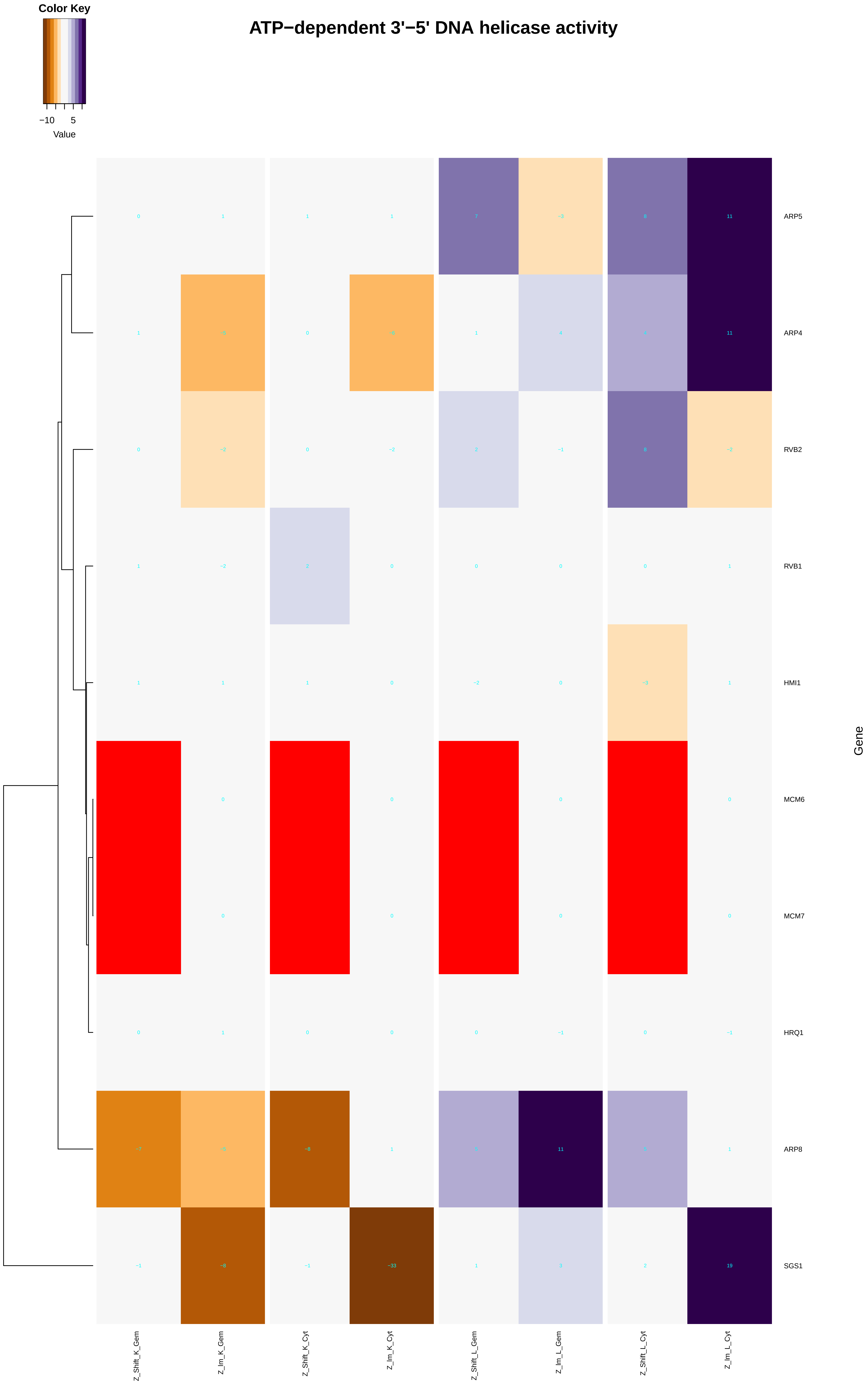

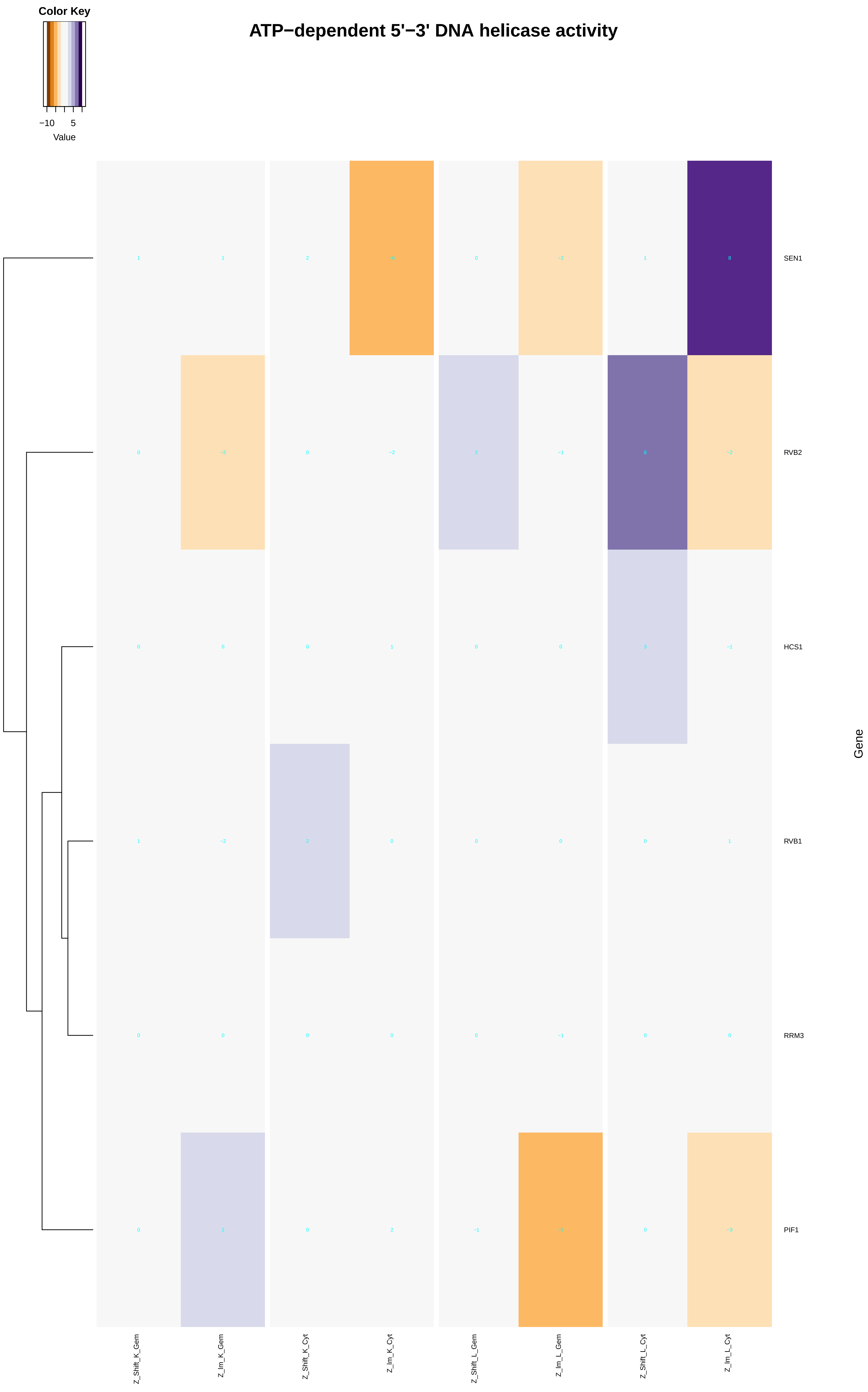
Color Key
−10
5
Value
ATP−dependent 5'−3' DNA helicase activity
SEN1
RVB2
HCS1
RVB1
RRM3
PIF1
Gene
Z_Shift_K_Gem
Z_Im_K_Gem
Z_Shift_K_Cyt
Z_Im_K_Cyt
Z_Shift_L_Gem
Z_Im_L_Gem
Z_Shift_L_Cyt
Z_Im_L_Cyt

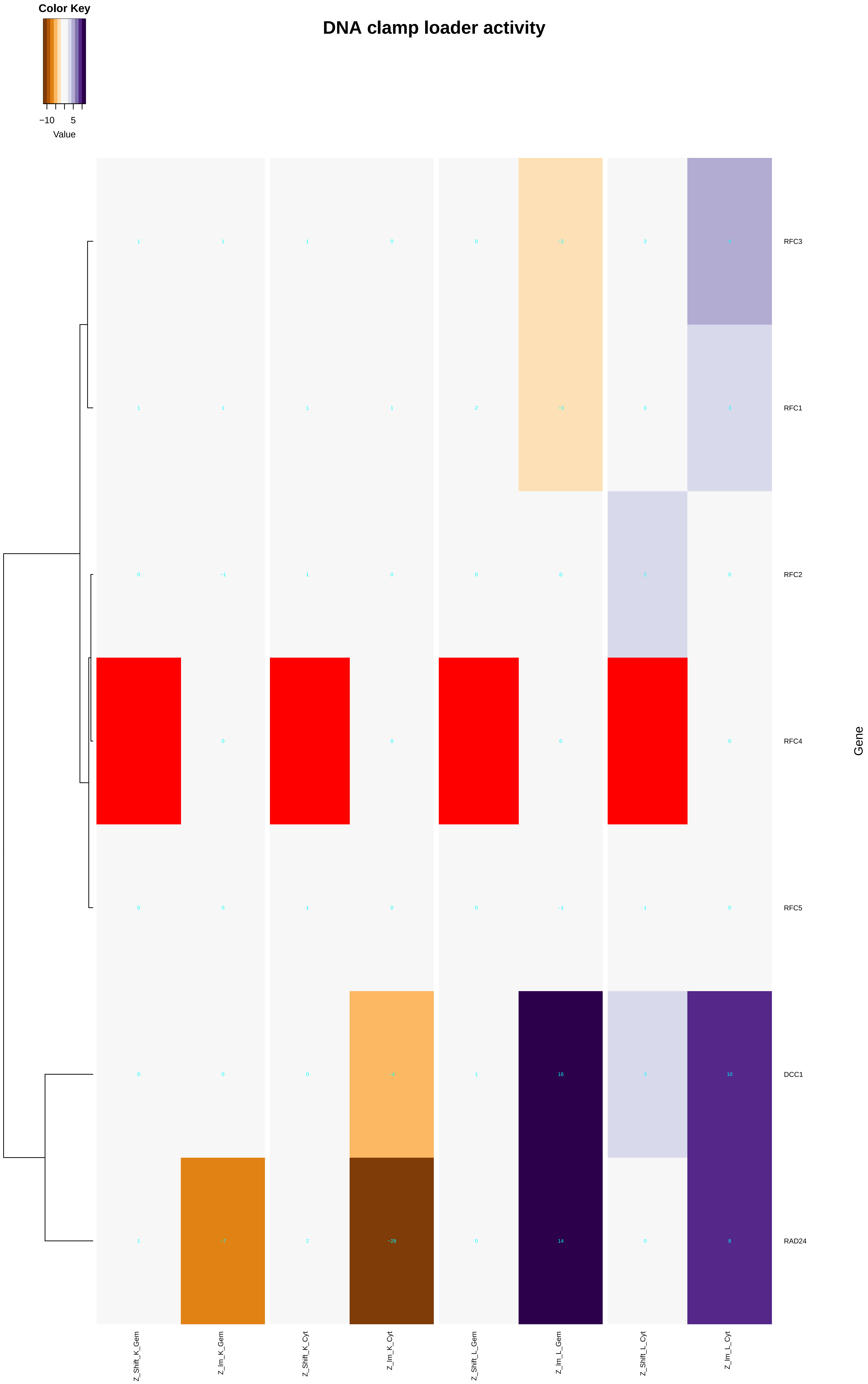
DNA clamp loader activity
Color Key
−10
5
Value
Gene
RFC3
RFC1
RFC2
RFC4
RFC5
DCC1
RAD24
Z_Shift_K_Gem
Z_Im_K_Gem
Z_Shift_K_Cyt
Z_Im_K_Cyt
Z_Shift_L_Gem
Z_Im_L_Gem
Z_Shift_L_Cyt
Z_Im_L_Cyt
1
1
1
0
0
−2
2
1
1
1
1
2
−3
0
3
0
−1
1
0
0
0
2
0
0
0
0
0
0
0
1
0
0
−1
1
0
0
0
0
1
16
3
10
1
−7
2
−28
0
14
0
8
